# Tumor-Associated Macrophage in Breast Tumor Microenvironment

**DOI:** 10.3390/ijms26135973

**Published:** 2025-06-21

**Authors:** Lingyao Ma, Yuexinzi Jin, Jian Xu, Jiexin Zhang

**Affiliations:** 1 Department of Laboratory Medicine, The First Affiliated Hospital of Nanjing Medical University, Nanjing 210029, China; 2 Branch of National Clinical Research Center for Laboratory Medicine, Nanjing 210029, China

**Keywords:** breast cancer, tumor microenvironment, tumor-associated macrophage, nanodrug delivery system

## Abstract

Breast cancer (BC) is the most common cancer in women worldwide. It is one of the main causes of cancer-related mortality. The breast tumor microenvironment (Br-TME) has emerged as an important factor related to BC development and prognosis. Tumor-associated macrophages (TAMs) are the main effector cells in the Br-TME; they play key roles in regulating angiogenesis, immunosuppression, metastasis, and chemoresistance in BC patients. In this review, we introduce the macrophage niche in the Br-TME, particularly emphasizing the origin of TAMs. Next, we summarize the typical pathways and molecular mechanisms of the interactions between TAMs and various other components in the Br-TME. Finally, we provide an overview of drugs that target TAMs and discuss the prevailing technologies for drug delivery in the context of BC treatment. Identification of the dynamic variations in tumor-promoting TAMs will help reveal the key links that drive BC progression. This review provides a theoretical basis for upcoming clinical trials that may substantially benefit patients.

## 1. Introduction

Breast cancer (BC) is the most commonly diagnosed malignant tumor in women and ranks second among causes of cancer-related death in women [1]. It is currently estimated that 1.6 million BC cases occur worldwide per year, affecting as many as one in eight women in high-income countries [2]. It is expected to account for 4.4 million diagnoses and 685,000 deaths in 2070 [3]. Approximately 18.4% of global BC cases occur in China [4]. Despite the considerable progress achieved with treatment protocols, metastatic breast cancer remains incurable [5].

In clinical practice, BC is recognized as a multifaceted disease [6]. Global gene expression profiling studies have classified BC into four subtypes by hierarchical clustering, namely, the luminal A subtype, luminal B subtype, human epidermal growth factor receptor 2 (HER2)-overexpression subtype, and triple-negative subtype (triple-negative breast cancer, TNBC) [7].

Both the luminal A subtype and luminal B subtype are positive for estrogen receptor (ER) and progesterone receptor (PR) expression. Luminal A tumors are negative for HER2 and have low expression of Ki-67, a nuclear marker of cell proliferation [8]. Luminal B tumors are divided into HER2-negative tumors and HER2-positive tumors with different levels of Ki-67 expression [7]. These two subtypes can be distinguished by the activation of two major biological processes: a proliferation/cell cycle-related pathway and a luminal/hormone-regulated pathway. Prat et al. analyzed the differences in the expression status of a panel of 50 genes between typical luminal A and luminal B subtypes [9]. They reported that the expression of genes involved in cell differentiation (e.g., *Krüppel-like factor 4* and *jun proto-oncogene*) and adhesion (e.g., *vinculin* and *type II collagen*) was upregulated in the luminal A subtype, whereas the expression of genes involved in the immune response [e.g., interleukin (IL)-2 receptors and CD86] and the cell cycle (e.g., cyclin B1 and radiation sensitivity 51 recombinase) was upregulated in the luminal B subtype [9]. The hallmark of precancerous lesions and BC is an increased proportion of ER^+^PR^+^ cells with increased Ki-67 expression [10]. Patients with high PR expression in luminal A tumors have a better baseline prognosis than those with luminal B tumors [11].

HER2 is encoded by the *erb-b2 receptor tyrosine kinase 2* (*ERBB2*) gene and is a transmembrane glycoprotein epidermal growth factor receptor (EGFR) with tyrosine kinase activity [12]. According to the guidelines of the American Society of Clinical Oncology and the College of American Pathologists in 2018, HER2 positivity is defined by an immunohistochemistry (IHC) score of 3 for HER2 and/or the in situ hybridization-based detection of HER2 amplification [13]. HER2-overexpressing BC is characterized by ER negativity and PR negativity [14]; it has a faster growth rate and a worse prognosis, with a 5-year survival rate of 50–60% [15]. Although HER2-targeting drugs (e.g., trastuzumab, lapatinib, and pertuzumab) can increase the survival rate, HER-2 downregulation and the existence of HER2 receptor variants [e.g., splicing variants of HER2 lacking exon 16 (HER2∆16)] may cause primary or acquired drug resistance [16].

TNBC often occurs in premenopausal women under 40 years of age, who account for approximately 15–20% of all BC patients [17]. TNBC is negative for ER, PR, and HER2 [18]. Compared with that of patients with other BC subtypes, the survival time of TNBC patients is significantly shorter; 40% of patients die within the first 5 years after diagnosis, and the recurrence rate after surgery is as high as 25% [19]. Jiang et al. analyzed clinical, genomic, and transcriptomic data from 465 patients with primary TNBC and proposed a Fudan classification system [20]. With this system, TNBC is classified into four transcriptome-based subtypes: luminal androgen receptor TNBC, immunomodulatory TNBC, basal-like immune-suppressed TNBC, and mesenchymal-like TNBC. Fan et al. conducted a single-center phase 2 study involving 139 untreated women with TNBC and confirmed a significant progression-free survival benefit of treatment based on the Fudan classification [21].

## 2. Macrophage Niche in the Breast Tumor Microenvironment

BC cells are surrounded by newly formed abnormal blood vessels, stromal cells, fibroblasts, endothelial cells, and innate and adaptive immune cells [22]. BC cells secrete soluble factors (e.g., growth factors and cytokines) to regulate the turnover of normal epithelial cells through interactions with the extracellular matrix (ECM) and cells in the breast tumor microenvironment (Br-TME) [23,24]. The crosstalk between BC cells and the Br-TME leads to a cascade of events that favor BC progression [25].

The Br-TME contains a variety of infiltrating immune cells; macrophages are the primary constituents related to innate immunity in the breast [26]. Higher expression levels of macrophage-related genes are directly correlated with more aggressive BC phenotypes [27]. Colony-stimulating factor 1 (CSF1) is a major growth factor that regulates the survival, differentiation, and proliferation of macrophages [28]. Cassetta et al. performed whole-tumor RNA sequencing for an independent cohort of 47 BC patients [29]. The results indicated that the CSF1^high^ group had a significantly greater macrophage signature score than the CSF1^mid^ group and the CSF1^low^ group did. The *sialic acid binding Ig-like lectin 1* (*SIGLEC1*) gene is one of the most upregulated genes in macrophages of the Br-TME and is correlated with the expression of the pan-macrophage marker CD163 [30]. Cassetta et al. also reported that SIGLEC1^+^ macrophages were enriched in basal subtypes rather than HER2-overexpressing and luminal subtypes [29]. These macrophages responded to BC by upregulating SIGLEC1 expression and producing tumor necrosis factor-α (TNF-α) to increase SIGLEC1 expression and vice versa. Both a high macrophage signature score and a higher proportion of SIGLEC1^+^ macrophages were significantly associated with shorter disease-specific survival in BC patients.

Changes in the composition of the ECM are associated with the increased migration of macrophages toward BC cells. In a cohort study of 278 BC patients, the deposition of hyaluronan (HA) was associated with increased macrophage counts and a poor prognosis, regardless of BC subtype [31]. An increase in ECM stiffness was positively correlated with the number of infiltrating macrophages in 20 BC patients [32]. Tenascin-C is an extracellular matrix glycoprotein that is transiently expressed in response to tissue injury to mediate fibrotic processes [33]. Tenascin-C is highly upregulated in primary and metastatic BC, and it is a predictor of metastatic recurrence and short overall survival [34]. Deligne et al. noted that macrophages in the Br-TME were abundant at sites where tenascin-C was overexpressed [35]. After treatment with a combination of an anti-tenascin-C antibody and anti-programmed cell death ligand 1 (PD-L1), macrophages translocated to the lesion edge from the stroma, which suppressed tumor growth and lung metastasis.

**Figure 1:**
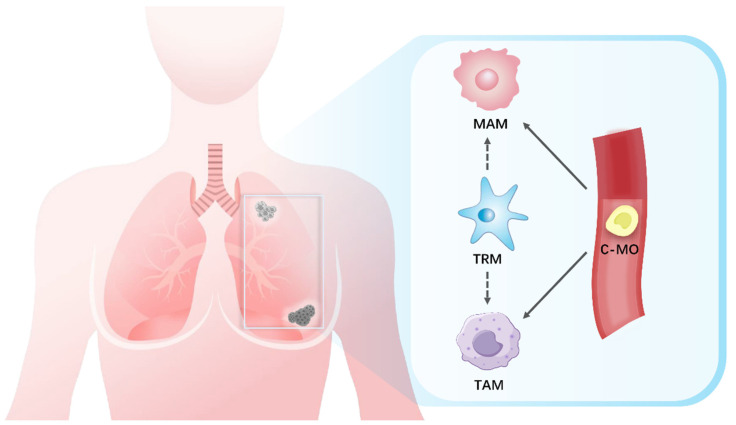
Macrophages in the Br-TME. The embryonic-derived tissue-resident macrophages (TRMs), the tumor-associated macrophages (TAMs), and the metastasis-associated macrophages (MAMs) are three groups of macrophages that are important components of the Br-TME. TRMs serve as a potential source for both TAMs and MAMs. Circulating classical monocytes (C-MOs) are necessary for TAM accumulation in the Br-TME via tumor cell-secreted factors (e.g., CCL 2, CCL18, CCL20, CSF1, and VEGF). The CCL2/CCR2 signaling axis drives the recruitment of C-MOs to premetastatic niches in distant tumor-challenged lesions and leads to MAM differentiation.

Macrophages constitute an intrinsically heterogeneous population characterized by significant plasticity (Figure 1).

The embryonic-derived tissue-resident macrophages (TRMs or Mϕ) infiltrating tumors are a source of macrophages, and they participate in cancer evolution and metastasis [36]. TRMs originate from the yolk sac during embryonic development and maintain self-sustaining populations to constitute the innate immune system [37,38]. In healthy breast tissues, TRMs are localized within the stroma of adipose tissue adjacent to the ductal epithelium [39]. TRMs participate in organizing the structure of terminal buds and extracellular matrix, and they exert anti-tumor immunity in a CSF1-dependent manner [40]. In fact, the CSF1 response has been found in many BC cases, and TRM reprogramming is associated with higher tumor proliferation rates and tumor grades [41]. Hypoxia and anaerobic glycolysis-induced changes in the hypoxia-inducible factor (HIF)-1α signaling pathway represent another reprogramming mechanism of TRMs that promotes BC [42,43]. Folate receptor beta (FOLR2) is a membrane protein involved in folate metabolism [44]. It has been reported that FOLR2^+^ TRMs induce naïve CD8^+^ T cells to transform into granzyme B^+^TNF-α^+^interferon-γ (IFN-γ)^+^ multifunctional effector cells in the Br-TME [45]. The F4/80^+^FOLR2^+^ subset is a favorable prognostic factor for hormone receptor-positive BC, and a high density of FOLR2^+^ TRMs was found to be directly correlated with an increased survival rate in BC patients [46]. However, Linde et al. found that CD206^hi^ intraepithelial TRMs upregulated the wingless-related integration site (Wnt)-1 through chemokine (C-C motif) ligand (CCL) 2 to disrupt E-cadherin junctions [47]. Stromal TRMs infiltrated into the epithelium, triggering early dissemination and subsequent metastasis in HER2^+^ BC.

In general, chronic inflammation in the TME promotes the polarization of macrophages into tumor-associated macrophages (TAMs) [48]. Clodronate liposomes (CLs) are inducers of macrophage apoptosis [49]. Hirano et al. used a green fluorescent protein-transgenic technology and injected the TNBC cell line E0771 into the fourth mammary gland fat pad (MGFP) of C57BL/6 J mice to assess macrophage distribution and tumor development [50]. In the CL-treated MGFP group, the number of TRMs was significantly reduced compared to the phosphate-buffered saline liposome-treated MGFP group, leading to decreased TAM infiltration, reduced tumor vessel density, and fewer distant metastases. This study suggested that TRMs are a major resource of TAMs and that TRMs contribute to the early development of TNBC. A recent study revealed that TAM heterogeneity in the Br-TME was driven by their territorial localization of pre-tumoral tissues [51]. The stromal/adipose TAMs expressed FOLR2 in both healthy and cancerous tissues, and advanced TAMs in the tumor nest were identified by the expressions of triggering receptors expressed on myeloid cells 2 and secreted phosphoprotein 1. The recruitment of circulating monocytes is also necessary for TAM accumulation in the Br-TME [52]. Peripheral monocytes are recruited to the Br-TME and differentiate into TAMs by factors such as CCL2, CCL18, CCL20, CSF1, and the vascular endothelial growth factor (VEGF), which are derived from both tumor cells and stromal cells [53,54].

The establishment of a premetastatic niche (PMN) represents a pivotal stage in the initiation and advancement of metastasis, which provides a conducive microenvironment for the settlement and colonization of disseminated tumor cells at distant metastatic sites [55]. Both monocyte-derived macrophages and TRMs contribute to metastasis and have been termed metastasis-associated macrophages (MAMs) [56,57]. When compared with TRMs and TAMs, MAMs expressed higher levels of vascular endothelial growth factor receptor 1 (VEGFR1, also known as FLT1) [58]. During angiogenesis, FLT1 binds the VEGF in a non-kinase-dependent manner with a 10-fold higher affinity than VEGFR2 [59]. Qian et al. constructed two independent mouse models of lineage FLT1 ablation and FLT1 kinase domain knockout to demonstrate that MAMs uniquely express FLT1 for BC cell survival after metastatic seeding by stimulating the downstream CSF1 autocrine signaling pathway [60]. In a BC metastasis mouse model, CCL2/C-C motif chemokine receptor (CCR) 2 axis induced the recruitment of circulating classical monocytes (C-MOs) into the PMN. Then, C-MOs preferentially migrated to the tumor-invaded lungs and differentiated into MAMs as early as 18 h [61]. Kitamura et al. used a monocyte tracking technology and revealed that C-MOs (F4/80^low^CD11b^+^Ly6C^+^) differentiated into a distinct F4/80^high^CD11b^high^Ly6C^high^ population of myeloid cells (called MAM precursor cells or MAMPCs) after migrating to the lungs with metastatic lesions [56]. MAMPCs and MAMs exhibited analogous mature macrophage markers, including CD14, CD36, CD64, and CD206. When compared to C-MOs, both MAMPCs and MAMs had upregulated expression levels of TAM signature genes. In a BC mouse model of lung metastasis, gene transcriptomic results demonstrated that newly recruited bone marrow-derived monocytes (BMDMs) were converted into precursor CD11b^hi^Ly6C^hi^ cells through the activation of the CCL2 receptor CCR2 [62]. This cell population produced CCL3 to promote the early accumulation of MAMs. The deletion of CCL3 or its receptor CCR1 in BMDMs could reverse this effect.

## 3. TAM Modulation by the Br-TME

### 3.1. Plasticity of TAMs

Depending on the signals in the microenvironment, TAMs in the Br-TME polarize into two distinct phenotypes: an antitumor subtype (lipopolysaccharide-induced macrophages, M1 TAMs) and a tumor-promoting subtype (IL-4-stimulated macrophages, M2 TAMs) [63]. In the early stages of BC, M1 TAMs activate antitumor immunity and suppress angiogenesis [64]. As tumors progress to advanced stages, the proportion of M2 TAMs increases, and M2 TAMs become the predominant subtype of TAMs in the Br-TME [65]. M2 TAMs are tumor-promoting factors that support BC progression by promoting angiogenesis, immune evasion, tumor stemness, tumor cell invasion, and metastasis [47,66,67].

M1 TAMs are immune effector cells with highly potent proinflammatory effects that release factors including but not limited to IL-6, IL-12, IL-23, and TNF-α to facilitate BC cell elimination [68,69]. Bernsmeier et al. reported that M1 TAMs that accumulated at sites of early oncogenesis produced nitric oxide (NO) and reactive oxygen species (ROS) to limit the proliferation of tumor cells [70]. The novel cysteine histone protease inhibitor GB111-NH2 inhibits BC development and induces the proliferation of M1 TAMs by increasing ROS levels [71]. M1 TAMs also directly engulf BC cells to present tumor antigens to activate cascades of antitumor immunity [72]. However, the prolonged activity of M1 TAMs fosters chronic inflammation and aggravates the genomic instability of malignant cells [73]. In this context, BC cells acquire the ability to induce M1 TAM repolarization toward the M2 phenotype [74,75].

M2 TAMs are rich in arginase-1 (ARG-1), mannose receptor (CD206), and the anti-inflammatory factor IL-10 [76]. M2 TAMs can be further classified into M2a, M2b, M2c, and M2d subsets [77]. The phenotype after polarization depends on interactions between specific ligands and their receptors, highlighting the dynamic status of the Br-TME [78]. The M2a subset (CD86^high^CD163^low^HLA-DR^high^) is induced by the T helper (Th) 2 cytokines IL-4 and IL-13 [79]. Little et al. reported that the concentrations of VEGF and CCL8 in the conditioned medium of M2a macrophages were significantly greater than those in the conditioned medium of M2b or M2c macrophages [80]. The M2b subset (IL-10^high^IL-12^low^CD86^+^MHCII^+^) is an intermediate subset between M1 and M2 TAMs [81]. Liu et al. established a BC xenograft model with continuous bevacizumab administration [82]. They revealed that the M2b subset was induced by the cross-communication of Fcγ receptors and Toll-like receptor 4 (TLR4), both of which were involved in acquired resistance to bevacizumab. Glucocorticoids and IL-10 induce the M2c subset (CD86^low^CD163^high^TLR1) [83]. They are closely related to the rapid proliferation and poor differentiation of primary ER^−^ BC [84]. The M2d subset (CD86^low^CD163^high^) is activated by IL-6 and induces angiogenesis and the growth of BC cell clusters [85].

**Figure 2:**
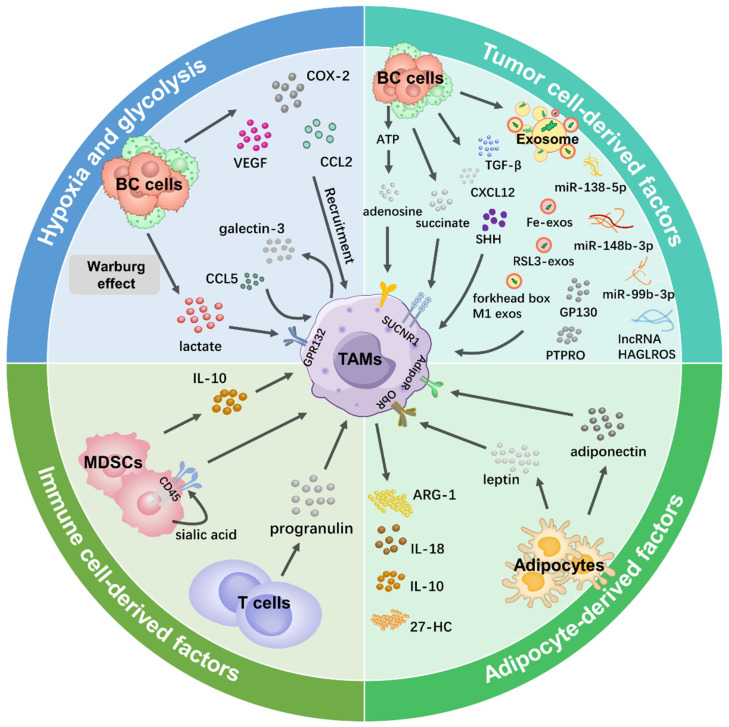
TAM modulation by the Br-TME. In four scenarios of the Br-TME, the primary factors that regulate TAMs are shown.

Here, we elucidated the regulatory mechanisms underlying the induction of M1 and M2 TAMs in the Br-TME, with a particular emphasis on M2 TAMs to explore how the regulation of these two macrophage phenotypes occurs in a reciprocal manner (Figure 2).

### 3.2. Hypoxia

Hypoxia is a prominent feature of solid tumors [86]. Long-term hypoxia is a major driving force for cancer progression [87,88]. Hypoxic regions of the Br-TME attract M2 TAMs by releasing the hypoxia-induced chemoattractants vascular endothelial growth factor A (VEGFA), angiopoietin (ANG) 2, CCL18, and prostate cancer-associated transcript 6 (PCAT6). HIF-1 and the nuclear factor kappa-light-chain enhancer of activated B cells (NF-κB) are activated in solid tumors in response to hypoxia to activate the expression of VEGF, COX-2, and monocyte chemoattractant protein 1 (MCP-1, also known as CCL2), which have been shown to recruit macrophages [89,90]. Notably, TAMs are distributed mainly in the hypoxic areas of solid tumors [91,92]. Wang et al. demonstrated that hypoxia induced the synthesis and secretion of galectin-3 from M2 TAMs in an autocrine manner to regulate NF-κB nuclear translocation [93]. By establishing an orthotopic syngeneic mouse model of mammary adenocarcinoma and a metastasis model by tail vein injection, they further confirmed that a galectin-3 inhibitor could suppress BC tumor growth and metastasis in hypoxic areas [94]. The exosomal circular RNA (circ)-0100519 was transcriptionally enhanced by HIF-1α and it was secreted from cancer cells [94]. After engulfed by TAMs, circ-0100519 suppressed nuclear factor-like 2 ubiquitination, leading to M2-TAM polarization.

TAMs synthesize two HIF isotypes in response to hypoxia [95]. HIF-1α is rapidly activated when acute and severe hypoxia (1–2% O_2_) occurs, and HIF-2α accumulates during prolonged and moderate hypoxia (<5% O_2_) [96]. HIF-1α and HIF-2α are both upregulated in TAMs within the hypoxic area of BC tumors [97]. Leek et al. reported that TAMs in the Br-TME preferentially migrated to hypoxic regions and strongly expressed HIF-2α, and these phenotypes were associated with high tumor vessel density, high tumor grade, and a poor prognosis in BC patients [98]. HIF-2α expression is an independent prognostic factor for recurrence-free survival and BC-specific survival [99]. Doedens et al. demonstrated in vitro that hypoxia enhanced TAM-mediated T cell suppression by altering HIF-1α expression [100]. Li et al. revealed that the HIF-1α inhibitor Lificiguat inhibited TNBC tumor growth and angiogenesis by transforming M2 TAMs into M1 TAMs in the Br-TME [101].

### 3.3. Glycolysis

The hypoxia-induced upregulation of glucose transporter-1 promotes glucose uptake for glycolysis to maintain TAM survival [102]. Tumor cells switch to anaerobic glycolysis (the “Warburg effect”) to reduce pyruvate oxidation and increase the excretion of lactate up to 40-fold via monocarboxylate transporter 4 [103]. Lactate and G protein-coupled receptor 132 (GPR132) are key molecules for TAM polarization and BC metastasis [104]. Chen et al. reported that BC cell-derived lactate activated GPR132 in macrophages for M2 phenotype polarization and that the alteration of GPR132 expression could inhibit lung metastasis and prolong the survival of BC patients [104]. Mu et al. reported that lactate stimulated M2 TAMs through activation of the extracellular signal-regulated protein kinase/signal transducer and activator of transcription 3 (STAT3) signaling pathway [105]. Lactate deprivation suppressed M2 phenotype polarization and led to devascularization, highlighting a new strategy for BC treatment. Lin et al. demonstrated that BC cell-derived lactate could also activate the neurogenic locus notch homolog signaling pathway to increase CCL5 secretion from M2 TAMs to form a positive feedback loop of glycolysis between M2 TAMs and BC cells [106]. Zinc finger E-box binding homeobox 1 (Zeb1) is a transcription factor that influences cellular homeostasis [107]. Zeb1 directly upregulates the expression of multiple glycolytic enzymes to promote the “Warburg effect” and BC invasiveness [108]. Jiang et al. reported that the ectopic expression of Zeb1 in BC cells resulted in the secretion of lactate to induce the TAM presentation of CD206, ARG-1, and IL-10 via the protein kinase A/cyclic AMP-responsive element-binding signaling pathway in a conditional knockout mouse model [109]. Niu et al. reported that high expressions of sodium/glucose transporter (SGLT1) in BC cells supported the maintenance of high levels of glycolysis and lactate metabolites, which stimulated the HIF-1α/STAT3 pathway to increase the number of M2 TAMs [110]. M2 TAMs upregulated SGLT1 expression in BC cells via the EGFR/phosphatidylinositol-3-kinase (PI3K)/protein kinase B (AKT) signaling pathway, leading to tamoxifen resistance and accelerating tumor growth both in vitro and in vivo, while the depletion of M2 TAMs induced the opposite effects.

### 3.4. Other Cell-Derived Factors in the Br-TME

#### 3.4.1. Tumor Cell-Derived Factors

CCL2 is widely expressed in solid tumor cells [111]. CCL2 expression levels are inversely correlated with the expression status of ER and PR [112]. CCL2 release from BC cells mediates the recruitment of peripheral monocytes to the Br-TME, where they differentiate toward the M2 phenotype [61,112]. The enhancer of zeste homolog 2 (EZH2) is a catalytic core subunit of polycomb repressive complex 2, and it interacts with the trimethylation of histone H3 lysine 27 to silence downstream tumor suppressor genes (such as *cyclin-dependent kinase inhibitor 1A* and *E-cadherin*) [113]. Archer et al. reported that the EZH2 knockdown in BC cells triggered CCL2 transcription and secretion to promote M2 TAM infiltration into the Br-TME [114]. Sushi domain containing 2 (SUSD2) is a transmembrane protein expressed on BC cells that promotes angiogenesis [115]. Hultgren et al. examined SUSD2 expression and TAM distribution in 175 BC patients by IHC staining and reported that at least 2-fold more M2 TAMs were in the SUSD2^high^ Br-TME [116]. SUSD2-expressing BC cells potentiated angiogenesis indirectly by CCL2 secretion, revealing a positive relationship between SUSD2^high^CCL2^high^ BC cells and increased proportions of M2 TAMs in BC tissues. Seoane et al. reported that the POU class 1 homeobox 1 transcription factor in BC cells mediated macrophage recruitment and polarization to M2 TAMs by releasing the chemokine (C-X-C motif) ligand (CXCL)12 [117]. Raschioni et al. demonstrated that activation of the C-X-C chemokine receptor 4 (CXCR4)/CXCL12 pathway was associated with the presence of M2 TAMs in primary BC of the luminal B subtype, maintaining the invasiveness of BC cells [118].

Succinate is an intermediate of the tricarboxylic acid cycle within mitochondria whose level is mainly controlled by the tumor suppressor succinate dehydrogenase (SDH) [119]. The gene mutation or activity inhibition of SDH causes the accumulation of succinate in BC cells [120]. Succinate is taken up by the succinate receptor (SUCNR1) on TAMs, and then M2 phenotype polarization is induced via the SUCNR1/PI3K/HIF-1α signaling pathway [121]. BC cells export ATP during hypoxia and chemotherapy [122,123]. Excessive ATP is rapidly degraded to adenosine [124]. Adenosine receptors are expressed on TAMs, and stimulation with adenosine induces M2 TAMs in the Br-TME [125].

Ma et al. demonstrated that BC cells secreted transforming growth factor β (TGF-β) to activate the TGF-β signaling pathway in TAMs, which significantly upregulated the expression of miR-182 in TAMs [126]. MiR-182 directly inhibited *TLR4* gene expression in TAMs to inactivate the downstream NF-κB signaling pathway, reducing the release of proinflammatory factors (e.g., TNF-α and IL-12) and promoting the production of the anti-inflammatory factor IL-10.

The hedgehog (Hh) pathway is one of the most active signaling pathways in cancer cells [127]. It is initiated by the binding of one of three sonic hedgehog ligands (SHHs), desert hedgehog, or Indian hedgehog to the patched-1 receptor, followed by smoothened (SMO) release and the transcriptional activation of glioma-associated oncogene homolog 1 (GLI 1) for cell proliferation, differentiation, EMT, and stem cell maintenance [128]. By administering the exogenous Hh/SMO inhibitor vismodegib to female BALB/c mice bearing orthotopic 4T1 tumors, Hanna et al. found that exogenous SHH protein upregulated M2 marker ARG-1 expression and that macrophages were polarized toward the immunosuppressive M2 TAM phenotype [129]. Treatment of TAMs with the small-molecule GLI 1 inhibitor GANT61, which could effectively inhibit the activity of the Hh signaling pathway in BC, restored M1 TAMs, as characterized by the upregulation of inducible nitric oxide synthase (iNOS) and TNF-α expression and the reduction in ARG-1 and CD206 expression, leading to CD8^+^ T cell trafficking back to the Br-TME where they exerted regulatory effects; these findings provide new ideas and directions for the clinical treatment of BC.

Snail family transcriptional repressor 1 (SNAIL1) is expressed in BC cells as well as in the Br-TME stroma [130]. SNAIL1 expression in primary BC is associated with a higher rate of recurrence, greater tumor aggressiveness, and worse outcomes [131]. Deletion of the *SNAIL1* gene significantly inhibits BC metastasis [132]. Brenot et al. established a MMTV-PyMT mouse model in which the *SNAIL1* gene was transfected into the TNBC cell line 4T1 [133]. They demonstrated that SNAIL1 inhibited the production of IL-1α, TNF-α, and the granulocyte-macrophage colony-stimulating factor to restrain the proinflammatory responses of TAMs and promote M2 TAM polarization.

Semaphorins (SEMAs) are a family of proteins that include the membrane-bound form of SEMA (e.g., SEMA7A, SEMA4C) and the secreted form of SEMA (e.g., SEMA3A, SEMA4D) [134]. Wallerius et al. reported that BC cell-derived SEMA3A worked through its receptor neuropilin 1 to suppress the proliferation of M2 TAMs in the Br-TME and that SEMA3A expression levels were correlated with the expression levels of genes characteristic of M1 TAMs, CD8^+^ T cells, and natural killer (NK) cells [135]. M2 TAMs are the main source of SEMA4D, which promotes endothelial cell migration, angiogenesis, and tumor growth in BC mice [136]. SEMA4C stimulates the recruitment of M2 TAMs and upregulates the expression of the proangiogenic factors ANG and CSF1 in the Br-TME [137]. Elder et al. demonstrated that SEMA7A overexpression in involutional breast epithelial cells stimulated the expression of myeloid-derived podoplanin (PDPN) on M2 TAMs, leading to the adhesion of PDPN-expressing M2 TAMs to lymphatic endothelial cells, lymphatic remodeling, and BC metastasis [138].

#### 3.4.2. Exosomes

Tumor-derived exosomes are considered important mediators of the crosstalk between BC cells and the Br-TME [139,140]. The role of tumor-derived exosomes in M2 TAM polarization has been emphasized in recent studies [141]. Ham et al. reported the presence of the IL-6 receptor glycoprotein 130 (GP130) in BC cell-derived exosomes [142]. GP130 phosphorylates STAT3 in macrophages, followed by STAT3 translocation to the nucleus to induce the transcription of IL-6, IL-10, CXCR4, and CCL2 to promote BMDM survival and TAM polarization to the M2 subtype. Piao et al. reported that TNBC-derived exosomes promoted the recruitment of CD206^+^ARG-1^+^ TAMs to create a favorable niche for lymph node metastasis [143]. Protein tyrosine phosphatase receptor type O (PTPRO) belongs to the receptor protein tyrosine phosphatase family [144]. PTPRO inhibits HER2-positive BC through dephosphorylation, leading to the dual effects of signaling suppression and the endosomal internalization of HER2 [145]. By generating stable PTPRO-overexpressing BC cells, Dong et al. reported that PTPRO in BC-derived exosomes induced M1 TAMs by mediating the dephosphorylation of STAT3 and STAT6 [146].

MiRNAs are a class of regulatory noncoding RNAs in exosomes [147]. MiRNAs regulate macrophage polarization in the Br-TME by regulating signaling pathways and the expression of multiple transcription factors [148]. Hao et al. isolated exosomes from the culture supernatant of BC cells and established an indirect coculture system of BC cells and TAMs and found that miR-148b-3p was overexpressed in BC cell-derived exosomes and induced M2 TAMs via the tuberous sclerosis complex 2/mTORC1 signaling pathway [149]. Xun et al. reported that lysine demethylase 6B (KDM6B) levels in THP-1 cells were significantly reduced after suspension coculture with the BC cell line MDA-MB-231 [150]. BC-derived exosomal miR-138-5p was shown to inhibit KDM6B expression in TAMs by binding to its 3′-untranslated region. The expression levels of the M1-related markers IL-6, TNF-α, and IL-1β were inhibited, and those of the M2-related markers CD163, ARG-1, IL-10, TGF-β, VEGFA, and CD163 were increased; these effects were partially reversed by treatment with a miR-138-5p inhibitor. Mao et al. used miRNA-seq technology to screen the differentially enriched miRNAs in the exosomes of drug-resistant and drug-susceptible BC cell lines [151]. They reported that BC cells and their exosomes contained high levels of miR-99b-3p and that these exosomes were taken up by TAMs in the Br-TME. MiR-99b-3p directly targeted the *serine/threonine-protein phosphatase 2A catalytic subunit alpha isoform* to induce the M2 polarization of TAMs, promoting the downstream phosphorylation of AKT/mTOR to stimulate the migration and paclitaxel resistance of BC cells. Meng et al. revealed that the lncRNA HAGLROS was highly expressed in the exosomes of BC cells and that this phenotype was related to a poor prognosis [152]. LncRNA HAGLROS, not only acted as competitive endogenous RNA to compete with miR-135b-3p, which increased the expression of its target gene *collagen type X alpha 1 chain*, but also phosphorylated STAT3 to induce M2 TAM polarization.

Ferroptosis is a novel form of iron-dependent cell death that plays a crucial role in the occurrence and development of BC [153]. Xiong et al. reported that short-term acidosis-induced ferroptosis in BC cells occurred in a zinc finger protein an1 type domain 5 (ZFAND5)/solute carrier family 3 member 2 (SLC3A2)-dependent manner [154]. Acidosis promoted the expression of ZFAND5, which promoted the ubiquitination process of SLC3A2 and inhibited its protein stability, thus decreasing glutathione synthesis in BC cells and inducing changes in mitochondrial morphology, resulting in the ferroptosis of BC cells. The ferroptosis of BC cells induces the increased expression of M1 markers (such as CD86 and iNOS), the secretion of proinflammatory cytokines (such as TNF-α and IL-12), and the decreased expression of M2 markers (such as CD206 and ARG-1) in TAMs, thereby promoting M1-type macrophage polarization and inhibiting tumor growth. Prognostic analysis revealed that high ZFAND5 expression or low SLC3A2 expression was associated with longer overall survival in BC patients. Yi et al. added the ferroptosis inducer erastin to the culture supernatant of the human MDA-MB-231 cell line and the mouse 4T1 cell line to increase Fe^2+^ levels and ROS production to induce ferroptosis in BC cells [155]. After treatment with erastin, the exosomes were collected and cocultured with macrophage lines (THP-1 cells and RAW 264.7 cells). The results indicated that ferroptosis-induced BC cell-derived exosomes (Fe-exos) significantly reduced the levels of the M2 markers CD206 and ARG-1 and increased the levels of the M1 markers CD68 and iNOS. Wang et al. generated exosomes derived from M1 TAMs and loaded them with a ferroptosis inducer, yielding RSL3-exos [156]. They reported that RSL3-exos spontaneously localized to and accumulated in the Br-TME and significantly promoted M1 TAMs by inhibiting the secretion of IL-10 and TGF-β, blocking the infiltration of regulatory T cells (Tregs), and suppressing fatty acid β-oxidation. Wang et al. reported that TNBC cells transmitted forkhead box M1-containing exosomes into TAMs for the transcriptional activation of indoleamine 2,3-dioxygenase 1, which increased the level of the metabolite quinolinic acid to impair ferroptosis [157].

#### 3.4.3. Adipocyte-Derived Factors

Breast adipose tissue is the main endocrine system of the mammary gland, where a variety of growth factors and enzymes are secreted to communicate with all components of the Br-TME [158]. Breast adipose tissue contains abundant infiltrated macrophages with a gene expression profile indicating inflammation, including CCR5, peroxisome proliferator-activated receptor (PPAR) α, IL-6, and IL-8 [159].

Adipocytes produce adiponectin (APN) in the Br-TME [160,161]. In response to APN, TAMs exhibit an anti-inflammatory M2 phenotype, and the production of proinflammatory cytokines is suppressed by the inhibition of the phosphorylation of inhibitors of NF-κB, c-Jun N-terminal kinase (JNK), p38, and STAT3 [160]. APN interacts with adiponectin receptors (AdipoRs) to promote M2 subtype polarization [162]. Tan et al. compared the signaling pathways stimulated by AdipoR1 and AdipoR2 [163]. AdipoR1 stimulated IL-10 production by activating the adenosine 5′-monophosphate (AMP)-activated protein kinase and p38 signaling pathways, whereas AdipoR2 regulated inflammation by activating COX-2 and PPARγ.

Leptin is a monocyte/macrophage chemoattractant [164]. It promotes tumor-promoting functions through binding with the canonical leptin receptor (ObR) [165]. Leptin exposure during TAM differentiation results in high levels of M2 TAM markers (ARG-1, IL-10, and CXCR4) and low levels of M1 TAM markers (iNOS and IL-12) [166]. Li et al. reported that leptin treatment increased the expression of a variety of cytokines in TAMs to promote the migration and invasion of BC cells; IL-18 regulation by the NF-κB/NF-κB1 signaling pathway was found to be the most significant change induced by this treatment [167].

Sphingomyelin (SM) is an important component of lipid rafts in the plasma membrane [168,169]. Sphingomyelin synthase 2 (SMS2) is a key enzyme that regulates the SM content [170]. Deng et al. reported that abundant SMS2 was associated with high expression levels of CD206 and ARG-1, large tumor sizes, and the development of lung metastasis [171]. SMS2 inhibitors or *SMS2* gene knockdown reduced M2 TAM polarization as well as tumor progression in a 4T1-TNBC mouse model.

Lipid accumulation is closely related to the tumor-promoting function of TAMs. Niu et al. demonstrated that caspase-1 induced the cleavage of aspartic acid at position 64 of PPARγ, which was then transported to mitochondria and interacted with medium-chain acyl-CoA dehydrogenase (MCAD) to inhibit fatty acid oxidation, leading to the accumulation of lipid droplets and M2 TAM polarization [172]. Caspase-1 inhibitors disrupt PPARγ/MCAD activity and repolarize TAMs rather than suppressing their activity. 27-Hydroxycholesterol (27-HC) is a metabolite of cholesterol. Shi et al. reported that cytochrome P450 family 27 subfamily A member 1, the synthetase of 27-HC, was more abundant in the THP-1 cell line than in BC cells and promoted the migration of CCR2- and CCR5-expressing monocytes to the Br-TME and their polarization into M2 TAMs [173]. M2 TAMs produced and released 27-HC, which not only promotes the proliferation, migration, and invasion of BC cells but also further promotes monocyte recruitment, ultimately contributing to the development of BC.

#### 3.4.4. TME Infiltration of Immune Cell-Derived Factors

Circulating myeloid-derived suppressor cells (MDSCs) are present in almost all solid tumors and are contributors to immunosuppression [174]. MDSCs are recruited to the Br-TME [175]. MDSCs trigger M2 TAM polarization in the Br-TME by secreting IL-10 [176]. In hypoxic regions, sialic acid from MDSCs induces the defragmentation of CD45 protein dimers to trigger tyrosine phosphatase activity in CD11b^+^Ly6C^hi^Ly6G^−^ monocytic MDSCs, followed by TAM differentiation through the STAT3 signaling pathway [177].

Progranulin (PGRN) is a growth factor secreted by T lymphocytes, and its overexpression has been identified in a variety of tumors [178]. Fang et al. treated a BC mouse model with PGRN, and the expression levels of the M2 TAM markers ARG-1 and CD206 were increased, with abundant PD-L1 on their surface [179]. This phenomenon might be reversed by treatment with a static STAT3 signaling inhibitor.

## 4. The Cancer-Promoting Functions of TAMs in the Br-TME

**Figure 3:**
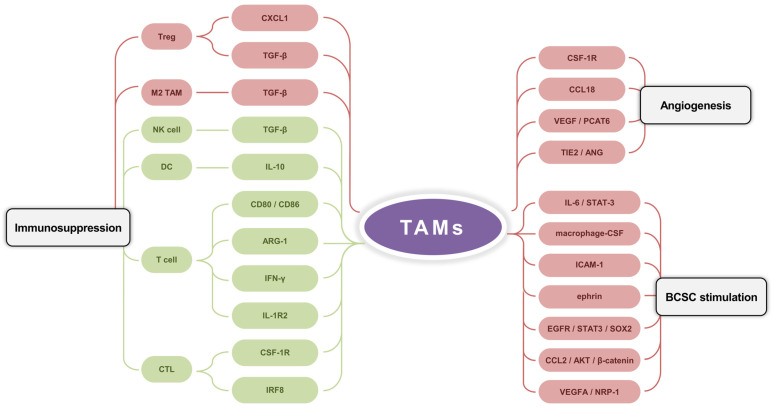
The cancer-promoting functions of TAMs in the Br-TME. TAMs facilitate angiogenesis by multiple factors and pathway cascades (e.g., CSF-1R, CCL18, VEGF/PCAT6, and TIE2/ANG). TAMs stimulate factors (e.g., macrophage-CSF, ICAM-1, and ephrin) and signaling pathways (e.g., IL-6/STAT-3, EGFR/STAT3/SOX2, CCL2/AKT/β-catenin, and VEGFA/NRP-1) to maintain the quantity and functionality of breast cancer stem cells. TAMs regulate the immune microenvironment by enhancing M2 TAM and Treg with TGF-β and CXCL1, and by inhibiting DC, NK cell, T cell, and CTL with TGF-β, IL-10, ARG-1, IFN-γ, IRF8, CD80/CD86, IL-1R2, and CSF-1R.

In the Br-TME, TAMs promote tumor progression through the angiogenesis, immunosuppression, and maintenance of BC cell stemness (Figure 3).

### 4.1. Angiogenesis

Angiogenesis is a key limiting step in the processes of tumor growth and metastasis. In the presence of M2 TAMs, angiogenesis is initiated [180]. Lin et al. generated a BC-sensitive transgenic mouse model and found that angiogenic transition and malignant transformation were delayed after knockdown of the CSF1 receptor (CSF-1R) on M2 TAMs [181]. Lin et al. used double IHC staining to assess the expression status of CCL18 in 80 tissue samples from BC patients [182]. The results indicated that the expression level of CCL18 was positively correlated with microvessel density in the Br-TME independent of VEGFR signaling pathway activity. When CCL18 was blocked with a neutralizing antibody, the ability of M2 TAMs to promote migration was suppressed, and angiogenesis was subsequently inhibited.

Dong et al. reported that M2 TAMs secreted the VEGF to stimulate PCAT6 expression and that PCAT6 promoted VEGFR2 expression in TNBC [183]. PCAT6 also participates in the VEGFR/AKT/mTOR signaling pathway to induce angiogenesis in the Br-TME [184]. Tyrosine kinases with immunoglobulin-like and EGF-like domain (TIE) receptors and their ANG ligands have been identified as a second vascular tissue-specific receptor tyrosine kinase system [185,186]. Palma et al. demonstrated that TIE2 was weakly expressed in circulating monocytes but was significantly upregulated during their homing to BC tumors and differentiation into perivascular macrophage subsets [187]. TIE2-expressing macrophages (TEMs) possess characteristics of M2 TAMs and promote angiogenesis and BC development [188]. Mazzieri et al. reported that the binding ability of TEMs to blood vessels was attenuated by the use of ANG blockers in a BC mouse model, followed by the downregulation of TIE2 expression on TEMs and the suppression of blood vessel perfusion in the Br-TME [189].

### 4.2. Immunosuppression

M2 TAMs in the Br-TME mainly exhibit immunosuppressive features [190]. The depletion of M2 TAMs using CSF-1R antagonists in resected breast tissue from 179 BC patients not only promoted the functions of cytotoxic T lymphocytes (CTLs) in antitumor immunity but also increased chemosensitivity [191]. The interaction between CD80/CD86 on M2 TAMs and the inhibitory T lymphocyte-associated protein 4 receptor on active T cells leads to cell cycle arrest in T cells [192]. M2 TAMs suppress the cytotoxicity of NK cells by releasing anti-inflammatory cytokines such as TGF-β [193]. Ruffell et al. compared the sorted epithelial cell populations and stromal cell populations in the Br-TME and revealed that M2 TAMs were the primary source of IL-10; these cells were found to inhibit IL-12 production by dendritic cells (DCs) to indirectly block the activation and proliferation of CD8^+^ CTLs [194]. Li et al. constructed a 4T1-Luc metastasis-tracking model of orthotropic BC and demonstrated that M2 TAM-mediated CXCL1 transcriptionally activates the NF-κB/forkhead box protein P3 signaling pathway to promote CD4^+^ T cell differentiation into Treg, followed by immune escape and lung metastasis [195]. TGF-β derived from M2 TAMs also facilitates Tregs infiltration into the mouse Br-TME and supports their ability to suppress CTLs [196]. Interferon regulatory factor 8 (IRF8) is a transcription factor required for the ability of M2 TAMs to present antigens to tumor cells. Nixon et al. used murine BC models with the myeloid cell-specific ablation of IRF8 and demonstrated that M2 TAMs caused the depletion of BC cell-reactive CTLs via IRF8 [197]. TAM-specific IRF8 deletion restored the CTL population and inhibited BC tumor growth.

L-arginine is a substrate of iNOS, a molecular marker of M1 TAMs, and it is responsible for the cytotoxic role of macrophages [192]. The increased levels of L-arginine caused the transition from glycolysis to oxidative phosphorylation in activated T cells and promoted the generation of central memory-like T cells with increased survival ability, increasing the antitumor activity of T cells [198]. The expression levels of ARG-1 are relatively high in BC patients [199]. ARG-1 hydrolyzes L-arginine [200]. ARG-1 catalyzes the conversion of L-arginine to urea and L-ornithine in TAMs, which deprives T cells of the raw materials essential for their proliferation [201,202]. L-ornithine promotes cell proliferation and repairs tissue injury through the generation of polyamines and collagen, both of which are highly immunosuppressive in the Br-TME [203].

M2 TAMs regulate the PD-1/PD-L1 axis [204]. M2 TAMs secrete IFN-γ to activate the signaling pathways of Janus kinase/STAT3 and PI3K/AKT to upregulate PD-L1 expression, inducing the apoptosis and immune anergy of tumor-specific T cells [205]. M2 TAMs enhance inhibitory activity through TGF-β-induced M2 TAM polarization and PD-L1 expression [206]. Xia et al. demonstrated that M2 TAMs promoted TNBC tumor growth via IL-1β-activated IL-1 receptor 2 (IL-1R2), whereas the IL-1R2 blockade strongly attenuated macrophage recruitment, M2 TAM polarization, and CD8^+^ T cell depletion, thereby reducing the tumor burden and prolonging the survival period [207]. Moreover, the use of an anti-IL-1R2 neutralizing antibody further increased the antitumor efficacy of a PD-1 antagonist.

### 4.3. Stimulation of Breast Cancer Stem Cells

Al-Hajj et al. isolated breast cancer stem cells (BCSCs) from primary or metastatic sites in nine patients and proposed that BCSCs serve as the origin of the tumorigenic population of cancer cells [208]. BCSCs have self-renewal and multidirectional differentiation abilities and proliferation potential. Moreover, BCSCs also possess the general characteristics of cancer cells, such as high tumorigenicity, high metastasis potential, and resistance to chemotherapy [209]. Radharani et al. induced RAW264.7 cell transformation into TAMs via culture with conditioned media from the murine 4T1 cell line; they reported that TAM-conditioned media enriched the BCSC populations and increased the expression levels of the BCSC-specific transcription factors *sex determining region Y-box 2* (*SOX-2*), *octamer-binding transcription factor-3*, *octamer-binding transcription factor-4*, and *nanog homeobox* [210]. They further demonstrated that IL-6 derived from TAMs played a key role in BCSC enrichment through the activation of STAT3. Lu et al. reported that M2 TAMs secreted macrophage-CSF, intercellular cell adhesion molecule-1, and ephrin to increase the survival, renewal, and tumorigenicity of BCSCs [211]. Yang et al. reported that M2 TAMs stimulated the BCSC phenotype and tumorigenesis by activating the EGFR/STAT3/SOX2 signaling pathway [212].

CD44^+^/CD24^−^ BCSCs are enriched in TNBC and are associated with the invasive behavior of TNBC [213]. β-catenin is a key factor in the Wnt signaling pathway [214]. Silencing β-catenin significantly reduces the proportion of CD44^+^/CD24^−^ BCSCs and the invasion ability of BC cells, which provides a new strategy for TNBC treatment [215]. Chen et al. demonstrated that M2 TAMs significantly promoted the stemness of the TNBC cell lines BT549 and HCC1937 by activating the CCL2/AKT/β-catenin signaling pathway [216]. Neuropilin-1 (NRP-1) is a transmembrane glycoprotein receptor that is highly expressed in BC and participates in cell proliferation, invasion, migration, and EMT [217,218]. Wang et al. reported that M2 TAMs secreted VEGFA to promote the stemness of TNBC cells and that the VEGFA/NRP-1 axis triggered the downstream Wnt/β-catenin signaling pathway to induce the BCSC phenotype [219].

## 5. Therapeutic Strategies and Prospects

TAMs are among the most important components of the Br-TME. Targeting TAMs is related to a series of emerging strategies for BC treatment, including limiting monocyte recruitment and TAM reprogramming [220].

Blockade of the CCL2/CCR2 axis markedly reduces the rate at which BC progresses by preventing the accumulation of TAMs in the Br-TME and increasing the antitumor efficacy of surrounding CD8^+^ T cells [192]. Nevertheless, several clinical trials have shown that CCL2 concentration is only transiently suppressed after the administration of a CCL2 neutralizing antibody (CNTO 888) and that CCL2 levels might exceed pretreatment baseline levels to cause the hypermetastasis of BC cells [221,222]. The blockade of CSF-1R has been shown to decrease M2 TAM infiltration and improve the chemotherapeutic response [191]. Compared with paclitaxel or carboplatin alone, the combination of CSF-1R signaling antagonists with paclitaxel or carboplatin exhibited enhanced antitumor efficacy and suppressed metastasis in preclinical BC models [223]. To date, the inhibition of CSF-1R has yet to translate into objective clinical responses. This is, in part, attributed to the elevated frequency of neutrophils and the depletion of anti-metastatic CD169^+^ lymph node macrophages [224,225].

Targeting immune inhibitory molecules on TAMs has also been considered as a strategy. RP-182 is a synthetic 10-mer amphipathic analog of host defense peptides that can selectively induce a conformational switch of the mannose receptor CD206 protein on the surface of M2 TAMs for phenotypic reprogramming [226]. CD40 agonists, PI3Kγ inhibitors, CD47 inhibitors, and histone deacetylase inhibitor subgroup II can suppress the growth of primary and metastatic murine BC through the conversion of M2 TAMs to M1 TAMs [203]. Bruton’s tyrosine kinase inhibitors, TLR agonists, STAT3 inhibitors, and inhibitors of interleukin-1 receptor antagonists have also been shown to block BC progression by interfering with signaling pathways related to M2 phenotype polarization [220]. The ROS regulator manganese(III) 5,10,15,20-tetrakis(1-ethyl-2-pyridinio) porphyrin pentachloride inhibits the IL-4-stimulated polarization of M2 TAMs by reducing STAT3 activation [227]. *T. mongolicum* extract has been used to treat breast inflammation and nodules. It remodels the Br-TME by inhibiting the IL-10/STAT3/PD-L1 immunosuppressive signaling pathway and by promoting the repolarization of M2 TAMs to suppress the proliferation, migration, and invasion of TNBC cells [228]. Poly (ADP–ribose) polymerase (PARP) is involved in the base excision repair process for DNA single-strand breaks. PARP inhibitors (PARPis) induce synthetic lethality in BC patients carrying loss-of-function mutations in *breast cancer susceptibility gene 1* (*BRCA1*) and *BRCA2* and are routinely used in the clinical treatment of metastatic BC [229]. However, Litton et al. reported that BC cells with the *BRCA1* gene defects strongly promoted M2 TAM infiltration and that the therapeutic response to PARPis was poor [230]. Wang et al. demonstrated that agonists of the small-molecule stimulator of interferon genes effectively reprogramed M2 TAMs into an antitumorigenic state, characterized by the induction of type I IFN responses and the expression of the costimulatory molecule CD86, and that the expression of CD86 could stimulate T cell cross-priming, increasing the sensitivity of BC cells with *BRCA1* and *BRCA2* mutations to PARPi treatment [231].

In recent years, many drugs have been developed to precisely regulate TAMs. However, the clinical applications remain limited due to the shortcomings of these agents, such as their poor solubility, rapid metabolism, nonselectivity, and off-target effects. It is also a challenge to modulate TAM phenotypes while maintaining their antitumor activities. Nanodrug delivery systems (NDDSs) have emerged as novel technologies for BC treatment. They offer advantages in enhancing the stability of macromolecules and promoting their cellular uptake within the Br-TME [232]. Li et al. devised a reactive oxygen species/glutathione dual-responsive drug conveyance platform (CUR/miR155@DssD-Hb NPs) to codeliver curcumin (CUR) and miR-155 [233]. CUR could promote the transformation of Tregs to type I helper T cells, induce the polarization of M2 TAMs toward M1 TAMs, and prevent the recruitment and aggregation of MDSCs in the Br-TME. The overexpression of miR-155 in M2 TAMs promoted their repolarization. Both in vitro and in vivo results revealed that the platform effectively reduced the proportions of viable BC cells and immunosuppressive cells (including MDSCs, Tregs, M2 TAMs, and exhausted T cells), stimulated DC maturation, and subsequently stimulated CD8^+^ T cell activation. To improve the chemotherapeutic effect in TNBC, Zhang et al. developed novel PANPs that were shelled with poly(lactic-co-glycolic acid) (PLGA) and loaded with perfluoropentane (PFP) for real-time tracking, paclitaxel, and an anti-miR-221 inhibitor [234]. TAMs were applied to internalize PANPs as RAW-PANPs, and they accumulated around BC cells to deliver PANPs. The results showed that RAW-PANPs inhibited both TNBC cell proliferation in vitro and BC tumor growth and progression in vivo, which had strong potential for treating TNBC patients presenting drug resistance. Clodronate is an important alternative for TAM removal and has been proven to effectively reduce tumor volume and weight in a 4T1 mouse model [235]. CL improves the pharmacokinetics of clodronate. Gu et al. reported that CL could deplete TAMs and significantly inhibit BC recurrence and bone metastasis [236]. Hydrazinocurcumin (HC) is a pyrazole derivative of curcumin that exerts antitumor effects through the repolarization of TAMs in the Br-TME [237]. Kumari et al. synthesized self-assembled amphiphilic PEGylated galactomannan (GM) nanoparticles loaded with HC (PSGM-HCNPs), and the results revealed that PSGM-HCNPs repolarized IL-4-stimulated RAW 264.7 cells by increasing ROS levels, decreasing CD206 and ARG-1 expression, and promoting proinflammatory cytokine secretion, thus reducing the tumor burden and prolonging the survival time of Ehrlich’s ascites carcinoma-bearing mice [238].

The recognition of TAM heterogeneity is becoming increasingly crucial for personalized BC therapy. However, the complexity of the regulatory mechanisms of macrophage polarization is still elusive, and there may be a variety of unknown signaling pathways and regulators; this complexity makes it difficult to identify the core regulatory factors of TAMs and develop new landmark drugs. It is imperative to carry out more comprehensive and in-depth research on the diverse interactions involving TAMs in the Br-TME.
